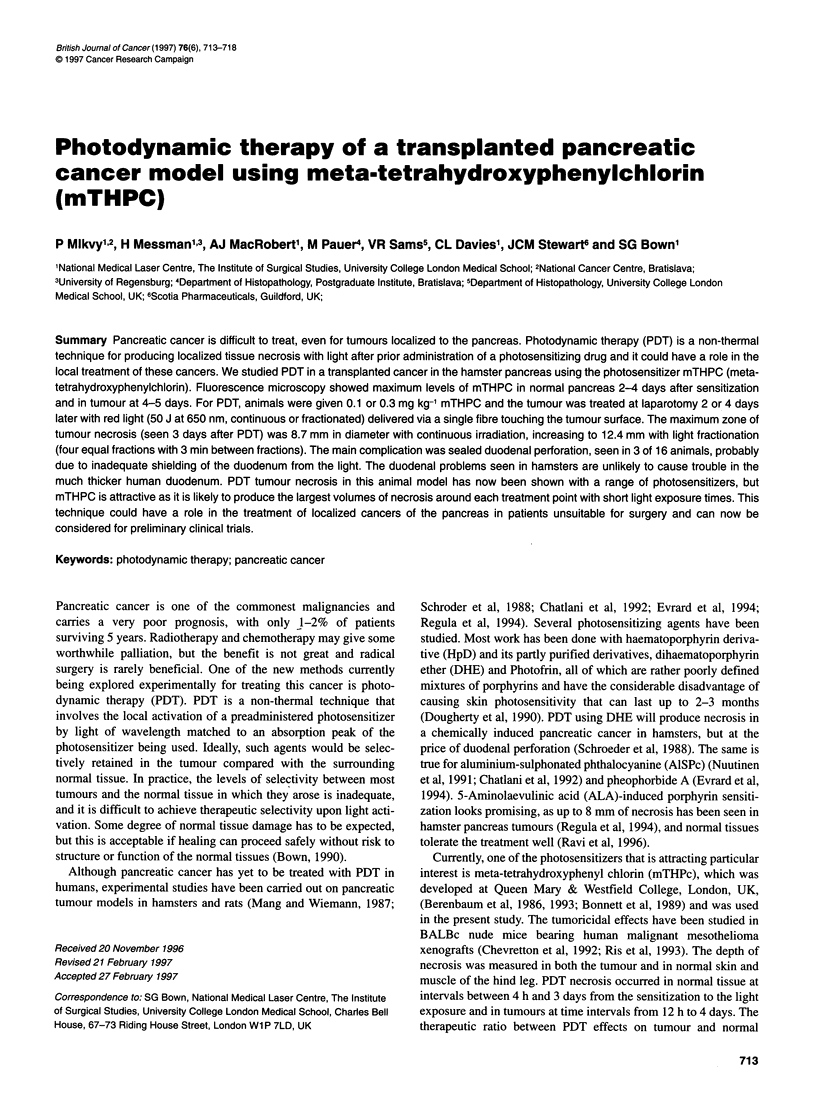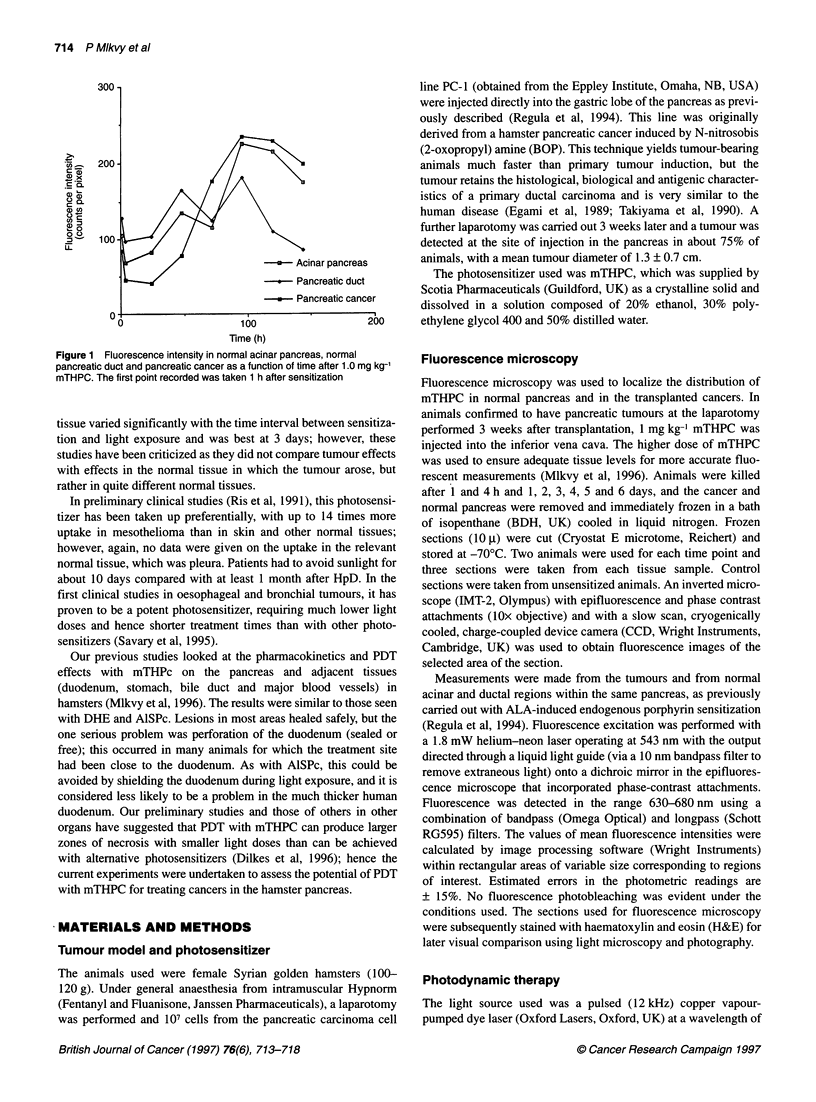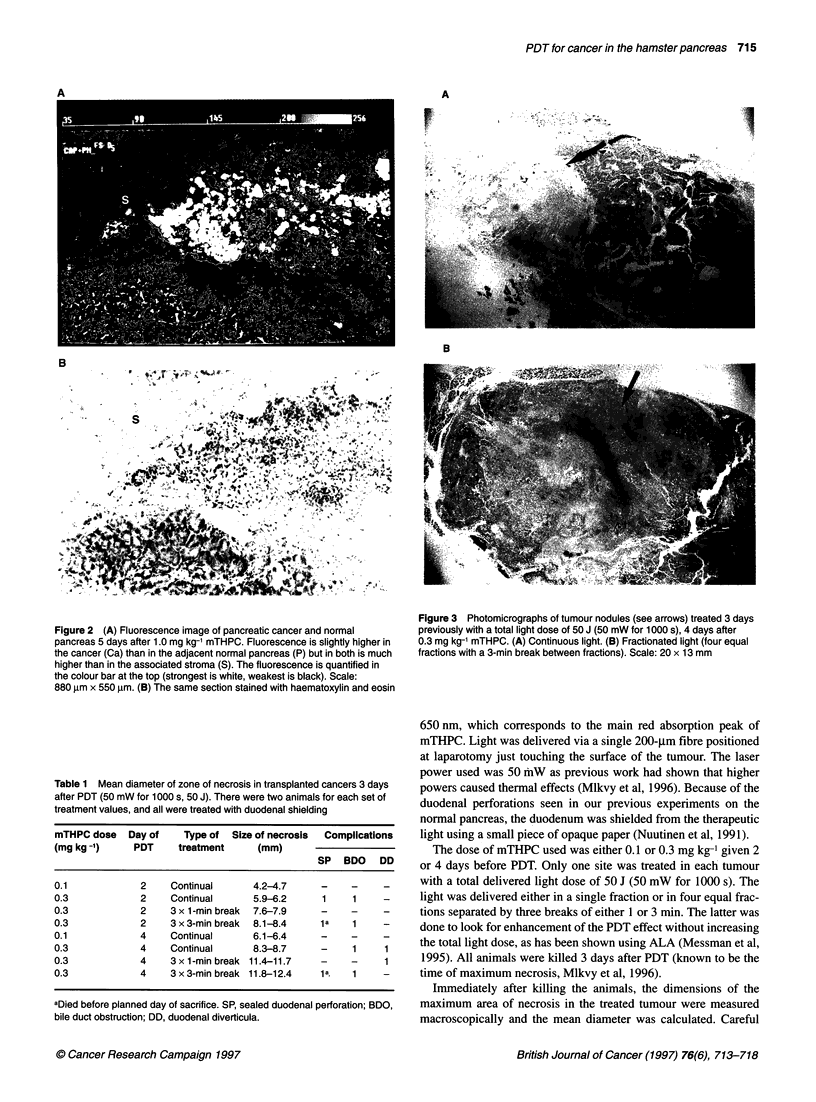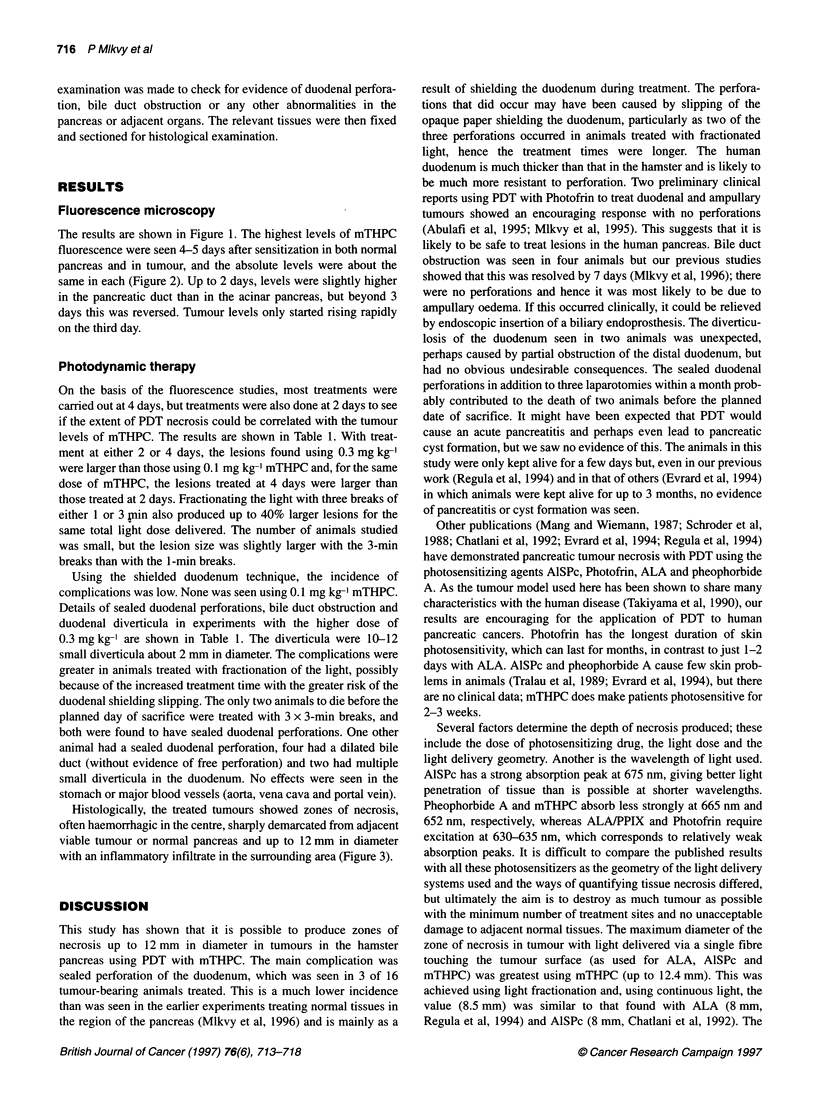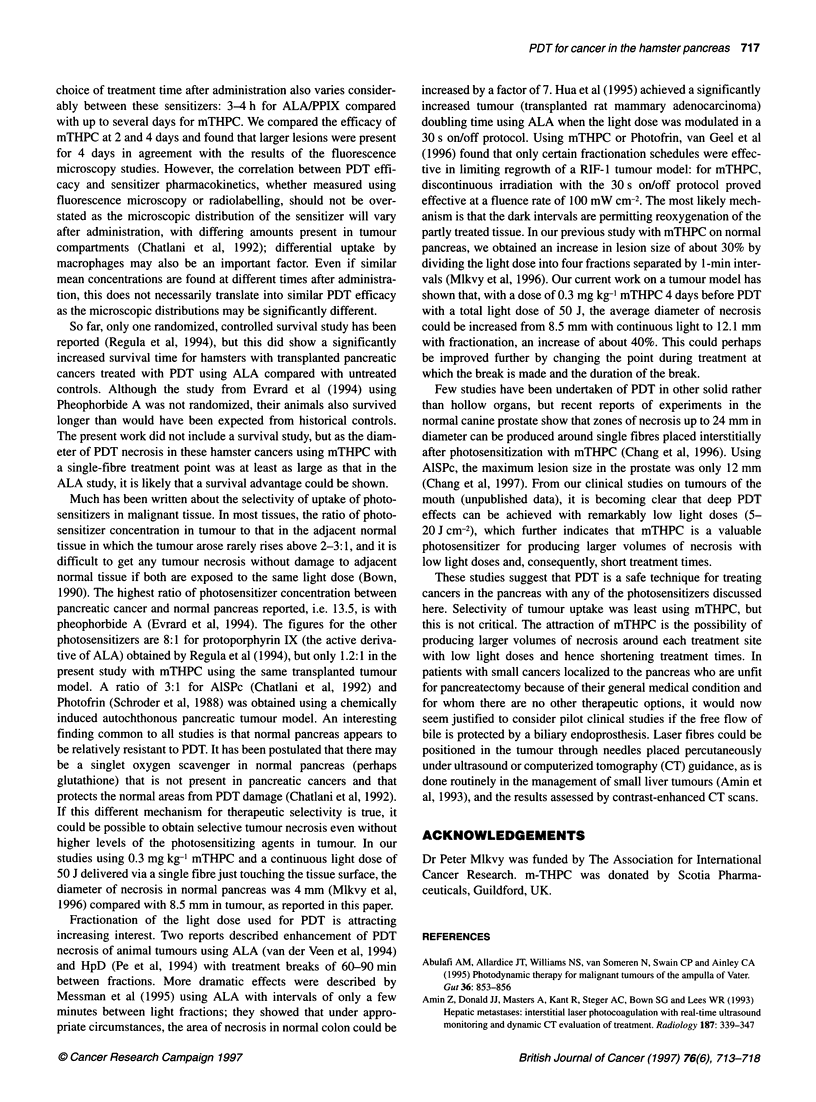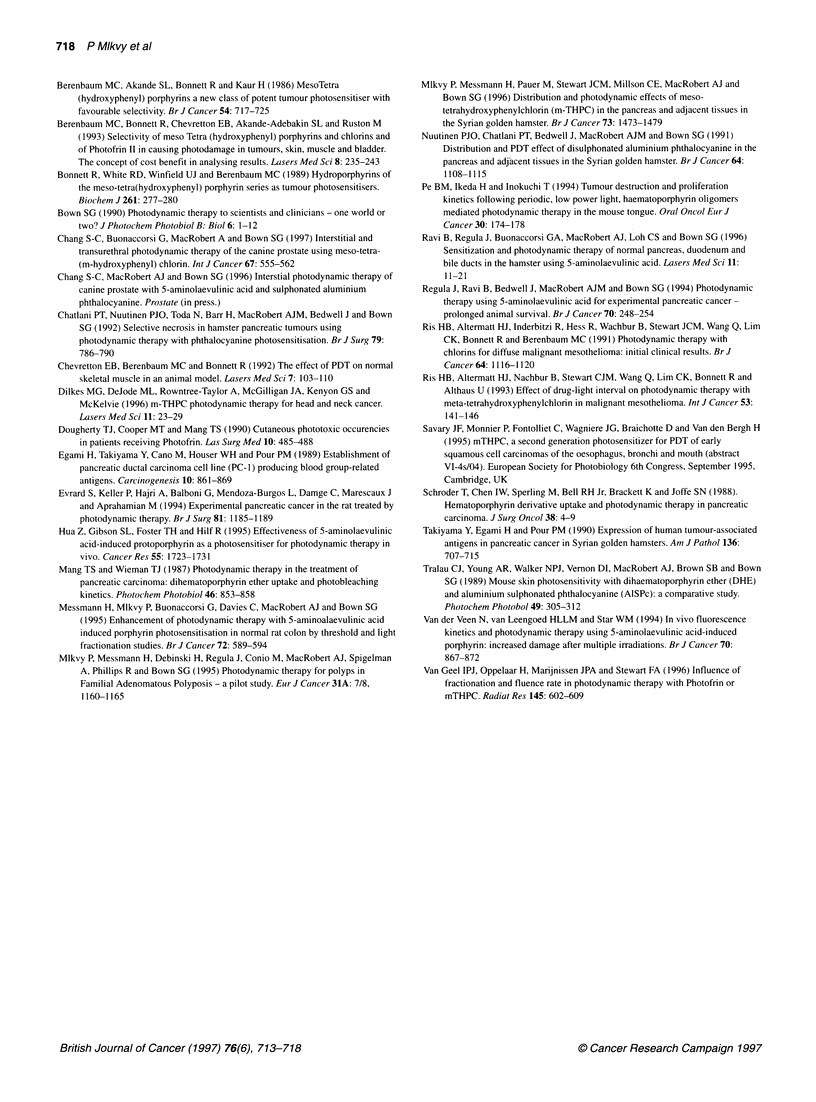# Photodynamic therapy of a transplanted pancreatic cancer model using meta-tetrahydroxyphenylchlorin (mTHPC).

**DOI:** 10.1038/bjc.1997.451

**Published:** 1997

**Authors:** P. Mikvy, H. Messman, A. J. MacRobert, M. Pauer, V. R. Sams, C. L. Davies, J. C. Stewart, S. G. Bown

**Affiliations:** National Medical Laser Centre, The Institute of Surgical Studies, University College London Medical School, UK.

## Abstract

**Images:**


British Journal of Cancer (1997) 76(6), 713–718
© 1997 Cancer Research Campaign

### Photodynamic therapy of a transplanted pancreatic cancer model using meta-tetrahydroxyphenylchlorin (mTHPC)

**P Mlkvy[1,2], H Messman[1,3], AJ MacRobert[1], M Pauer[4], VR Sams[5], CL Davies[1], JCM Stewart[6] and SG Bown[1]**

[1]National Medical Laser Centre, The Institute of Surgical Studies, University College London Medical School; [2]National Cancer Centre, Bratislava; [3]University of Regensburg; [4]Department of Histopathology, Postgraduate Institute, Bratislava; [5]Department of Histopathology, University College London Medical School, UK; [6]Scotia Pharmaceuticals, Guildford, UK;

**Summary** Pancreatic cancer is difficult to treat, even for tumours localized to the pancreas. Photodynamic therapy (PDT) is a non-thermal technique for producing localized tissue necrosis with light after prior administration of a photosensitizing drug and it could have a role in the local treatment of these cancers. We studied PDT in a transplanted cancer in the hamster pancreas using the photosensitizer mTHPC (meta-tetrahydroxyphenylchlorin). Fluorescence microscopy showed maximum levels of mTHPC in normal pancreas 2–4 days after sensitization and in tumour at 4–5 days. For PDT, animals were given 0.1 or 0.3 mg $kg^{-1}$ mTHPC and the tumour was treated at laparotomy 2 or 4 days later with red light (50 J at 650 nm, continuous or fractionated) delivered via a single fibre touching the tumour surface. The maximum zone of tumour necrosis (seen 3 days after PDT) was 8.7 mm in diameter with continuous irradiation, increasing to 12.4 mm with light fractionation (four equal fractions with 3 min between fractions). The main complication was sealed duodenal perforation, seen in 3 of 16 animals, probably due to inadequate shielding of the duodenum from the light. The duodenal problems seen in hamsters are unlikely to cause trouble in the much thicker human duodenum. PDT tumour necrosis in this animal model has now been shown with a range of photosensitizers, but mTHPC is attractive as it is likely to produce the largest volumes of necrosis around each treatment point with short light exposure times. This technique could have a role in the treatment of localized cancers of the pancreas in patients unsuitable for surgery and can now be considered for preliminary clinical trials.

**Keywords:** photodynamic therapy; pancreatic cancer

Pancreatic cancer is one of the commonest malignancies and carries a very poor prognosis, with only 1–2% of patients surviving 5 years. Radiotherapy and chemotherapy may give some worthwhile palliation, but the benefit is not great and radical surgery is rarely beneficial. One of the new methods currently being explored experimentally for treating this cancer is photodynamic therapy (PDT). PDT is a non-thermal technique that involves the local activation of a preadministered photosensitizer by light of wavelength matched to an absorption peak of the photosensitizer being used. Ideally, such agents would be selectively retained in the tumour compared with the surrounding normal tissue. In practice, the levels of selectivity between most tumours and the normal tissue in which they arose is inadequate, and it is difficult to achieve therapeutic selectivity upon light activation. Some degree of normal tissue damage has to be expected, but this is acceptable if healing can proceed safely without risk to structure or function of the normal tissues (Bown, 1990).

Although pancreatic cancer has yet to be treated with PDT in humans, experimental studies have been carried out on pancreatic tumour models in hamsters and rats (Mang and Wiemann, 1987; Schroder et al, 1988; Chatlani et al, 1992; Evrard et al, 1994; Regula et al, 1994). Several photosensitizing agents have been studied. Most work has been done with haematoporphyrin derivative (HpD) and its partly purified derivatives, dihaematoporphyrin ether (DHE) and Photofrin, all of which are rather poorly defined mixtures of porphyrins and have the considerable disadvantage of causing skin photosensitivity that can last up to 2–3 months (Dougherty et al, 1990). PDT using DHE will produce necrosis in a chemically induced pancreatic cancer in hamsters, but at the price of duodenal perforation (Schroeder et al, 1988). The same is true for aluminium-sulphonated phthalocyanine (AlSPc) (Nuutinen et al, 1991; Chatlani et al, 1992) and pheophorbide A (Evrard et al, 1994). 5-Aminolaevulinic acid (ALA)-induced porphyrin sensitization looks promising, as up to 8 mm of necrosis has been seen in hamster pancreas tumours (Regula et al, 1994), and normal tissues tolerate the treatment well (Ravi et al, 1996).

Currently, one of the photosensitizers that is attracting particular interest is meta-tetrahydroxyphenyl chlorin (mTHPc), which was developed at Queen Mary & Westfield College, London, UK, (Berenbaum et al, 1986, 1993; Bonnett et al, 1989) and was used in the present study. The tumoricidal effects have been studied in BALBc nude mice bearing human malignant mesothelioma xenografts (Chevretton et al, 1992; Ris et al, 1993). The depth of necrosis was measured in both the tumour and in normal skin and muscle of the hind leg. PDT necrosis occurred in normal tissue at intervals between 4 h and 3 days from the sensitization to the light exposure and in tumours at time intervals from 12 h to 4 days. The therapeutic ratio between PDT effects on tumour and normal

*Received 20 November 1996*
*Revised 21 February 1997*
*Accepted 27 February 1997*

*Correspondence to:* SG Bown, National Medical Laser Centre, The Institute of Surgical Studies, University College London Medical School, Charles Bell House, 67–73 Riding House Street, London W1P 7LD, UK

**Figure 1** Fluorescence intensity in normal acinar pancreas, normal pancreatic duct and pancreatic cancer as a function of time after 1.0 mg $kg^{-1}$ mTHPC. The first point recorded was taken 1 h after sensitization tissue varied significantly with the time interval between sensitization and light exposure and was best at 3 days; however, these studies have been criticized as they did not compare tumour effects with effects in the normal tissue in which the tumour arose, but rather in quite different normal tissues.

In preliminary clinical studies (Ris et al, 1991), this photosensitizer has been taken up preferentially, with up to 14 times more uptake in mesothelioma than in skin and other normal tissues; however, again, no data were given on the uptake in the relevant normal tissue, which was pleura. Patients had to avoid sunlight for about 10 days compared with at least 1 month after HpD. In the first clinical studies in oesophageal and bronchial tumours, it has proven to be a potent photosensitizer, requiring much lower light doses and hence shorter treatment times than with other photosensitizers (Savary et al, 1995).

Our previous studies looked at the pharmacokinetics and PDT effects with mTHPc on the pancreas and adjacent tissues (duodenum, stomach, bile duct and major blood vessels) in hamsters (Mlkvy et al, 1996). The results were similar to those seen with DHE and AlSPc. Lesions in most areas healed safely, but the one serious problem was perforation of the duodenum (sealed or free); this occurred in many animals for which the treatment site had been close to the duodenum. As with AlSPc, this could be avoided by shielding the duodenum during light exposure, and it is considered less likely to be a problem in the much thicker human duodenum. Our preliminary studies and those of others in other organs have suggested that PDT with mTHPC can produce larger zones of necrosis with smaller light doses than can be achieved with alternative photosensitizers (Dilkes et al, 1996); hence the current experiments were undertaken to assess the potential of PDT with mTHPC for treating cancers in the hamster pancreas.

### MATERIALS AND METHODS

### Tumour model and photosensitizer

The animals used were female Syrian golden hamsters (100–120 g). Under general anaesthesia from intramuscular Hypnorm (Fentanyl and Fluanisone, Janssen Pharmaceuticals), a laparotomy was performed and $10^7$ cells from the pancreatic carcinoma cell line PC-1 (obtained from the Eppley Institute, Omaha, NB, USA) were injected directly into the gastric lobe of the pancreas as previously described (Regula et al, 1994). This line was originally derived from a hamster pancreatic cancer induced by N-nitrosobis (2-oxopropyl) amine (BOP). This technique yields tumour-bearing animals much faster than primary tumour induction, but the tumour retains the histological, biological and antigenic characteristics of a primary ductal carcinoma and is very similar to the human disease (Egami et al, 1989; Takiyama et al, 1990). A further laparotomy was carried out 3 weeks later and a tumour was detected at the site of injection in the pancreas in about 75% of animals, with a mean tumour diameter of 1.3 ± 0.7 cm.

The photosensitizer used was mTHPC, which was supplied by Scotia Pharmaceuticals (Guildford, UK) as a crystalline solid and dissolved in a solution composed of 20% ethanol, 30% polyethylene glycol 400 and 50% distilled water.

### Fluorescence microscopy

Fluorescence microscopy was used to localize the distribution of mTHPC in normal pancreas and in the transplanted cancers. In animals confirmed to have pancreatic tumours at the laparotomy performed 3 weeks after transplantation, 1 mg $kg^{-1}$ mTHPC was injected into the inferior vena cava. The higher dose of mTHPC was used to ensure adequate tissue levels for more accurate fluorescent measurements (Mlkvy et al, 1996). Animals were killed after 1 and 4 h and 1, 2, 3, 4, 5 and 6 days, and the cancer and normal pancreas were removed and immediately frozen in a bath of isopenthane (BDH, UK) cooled in liquid nitrogen. Frozen sections (10 µ) were cut (Cryostat E microtome, Reichert) and stored at –70°C. Two animals were used for each time point and three sections were taken from each tissue sample. Control sections were taken from unsensitized animals. An inverted microscope (IMT-2, Olympus) with epifluorescence and phase contrast attachments (10× objective) and with a slow scan, cryogenically cooled, charge-coupled device camera (CCD, Wright Instruments, Cambridge, UK) was used to obtain fluorescence images of the selected area of the section.

Measurements were made from the tumours and from normal acinar and ductal regions within the same pancreas, as previously carried out with ALA-induced endogenous porphyrin sensitization (Regula et al, 1994). Fluorescence excitation was performed with a 1.8 mW helium–neon laser operating at 543 nm with the output directed through a liquid light guide (via a 10 nm bandpass filter to remove extraneous light) onto a dichroic mirror in the epifluorescence microscope that incorporated phase-contrast attachments. Fluorescence was detected in the range 630–680 nm using a combination of bandpass (Omega Optical) and longpass (Schott RG595) filters. The values of mean fluorescence intensities were calculated by image processing software (Wright Instruments) within rectangular areas of variable size corresponding to regions of interest. Estimated errors in the photometric readings are ± 15%. No fluorescence photobleaching was evident under the conditions used. The sections used for fluorescence microscopy were subsequently stained with haematoxylin and eosin (H&E) for later visual comparison using light microscopy and photography.

### Photodynamic therapy

The light source used was a pulsed (12 kHz) copper vapour-pumped dye laser (Oxford Lasers, Oxford, UK) at a wavelength of

 © Cancer Research Campaign 1997

**Figure 2** (**A**) Fluorescence image of pancreatic cancer and normal pancreas 5 days after 1.0 mg kg$^{-1}$ mTHPC. Fluorescence is slightly higher in the cancer (Ca) than in the adjacent normal pancreas (P) but in both is much higher than in the associated stroma (S). The fluorescence is quantified in the colour bar at the top (strongest is white, weakest is black). Scale: 880 μm × 550 μm. (**B**) The same section stained with haematoxylin and eosin

**Figure 3** Photomicrographs of tumour nodules (see arrows) treated 3 days previously with a total light dose of 50 J (50 mW for 1000 s), 4 days after 0.3 mg kg$^{-1}$ mTHPC. (**A**) Continuous light. (**B**) Fractionated light (four equal fractions with a 3-min break between fractions). Scale: 20 × 13 mm

**Table 1** Mean diameter of zone of necrosis in transplanted cancers 3 days after PDT (50 mW for 1000 s, 50 J). There were two animals for each set of treatment values, and all were treated with duodenal shielding

| mTHPC dose (mg kg$^{-1}$) | Day of PDT | Type of treatment | Size of necrosis (mm) | Complications | | |
|---|---|---|---|---|---|---|
| | | | | SP | BDO | DD |
| 0.1 | 2 | Continual | 4.2–4.7 | – | – | – |
| 0.3 | 2 | Continual | 5.9–6.2 | 1 | 1 | – |
| 0.3 | 2 | 3 × 1-min break | 7.6–7.9 | – | – | – |
| 0.3 | 2 | 3 × 3-min break | 8.1–8.4 | 1[a] | 1 | – |
| 0.1 | 4 | Continual | 6.1–6.4 | – | – | – |
| 0.3 | 4 | Continual | 8.3–8.7 | – | 1 | 1 |
| 0.3 | 4 | 3 × 1-min break | 11.4–11.7 | – | – | 1 |
| 0.3 | 4 | 3 × 3-min break | 11.8–12.4 | 1[a]. | 1 | – |

[a]Died before planned day of sacrifice. SP, sealed duodenal perforation; BDO, bile duct obstruction; DD, duodenal diverticula.

650 nm, which corresponds to the main red absorption peak of mTHPC. Light was delivered via a single 200-μm fibre positioned at laparotomy just touching the surface of the tumour. The laser power used was 50 mW as previous work had shown that higher powers caused thermal effects (Mlkvy et al, 1996). Because of the duodenal perforations seen in our previous experiments on the normal pancreas, the duodenum was shielded from the therapeutic light using a small piece of opaque paper (Nuutinen et al, 1991).

The dose of mTHPC used was either 0.1 or 0.3 mg kg$^{-1}$ given 2 or 4 days before PDT. Only one site was treated in each tumour with a total delivered light dose of 50 J (50 mW for 1000 s). The light was delivered either in a single fraction or in four equal fractions separated by three breaks of either 1 or 3 min. The latter was done to look for enhancement of the PDT effect without increasing the total light dose, as has been shown using ALA (Messman et al, 1995). All animals were killed 3 days after PDT (known to be the time of maximum necrosis, Mlkvy et al, 1996).

Immediately after killing the animals, the dimensions of the maximum area of necrosis in the treated tumour were measured macroscopically and the mean diameter was calculated. Careful

© *Cancer Research Campaign 1997*

examination was made to check for evidence of duodenal perforation, bile duct obstruction or any other abnormalities in the pancreas or adjacent organs. The relevant tissues were then fixed and sectioned for histological examination.

### RESULTS

### Fluorescence microscopy

The results are shown in Figure 1. The highest levels of mTHPC fluorescence were seen 4–5 days after sensitization in both normal pancreas and in tumour, and the absolute levels were about the same in each (Figure 2). Up to 2 days, levels were slightly higher in the pancreatic duct than in the acinar pancreas, but beyond 3 days this was reversed. Tumour levels only started rising rapidly on the third day.

### Photodynamic therapy

On the basis of the fluorescence studies, most treatments were carried out at 4 days, but treatments were also done at 2 days to see if the extent of PDT necrosis could be correlated with the tumour levels of mTHPC. The results are shown in Table 1. With treatment at either 2 or 4 days, the lesions found using 0.3 mg $kg^{-1}$ were larger than those using 0.1 mg $kg^{-1}$ mTHPC and, for the same dose of mTHPC, the lesions treated at 4 days were larger than those treated at 2 days. Fractionating the light with three breaks of either 1 or 3 min also produced up to 40% larger lesions for the same total light dose delivered. The number of animals studied was small, but the lesion size was slightly larger with the 3-min breaks than with the 1-min breaks.

Using the shielded duodenum technique, the incidence of complications was low. None was seen using 0.1 mg $kg^{-1}$ mTHPC. Details of sealed duodenal perforations, bile duct obstruction and duodenal diverticula in experiments with the higher dose of 0.3 mg $kg^{-1}$ are shown in Table 1. The diverticula were 10–12 small diverticula about 2 mm in diameter. The complications were greater in animals treated with fractionation of the light, possibly because of the increased treatment time with the greater risk of the duodenal shielding slipping. The only two animals to die before the planned day of sacrifice were treated with 3 × 3-min breaks, and both were found to have sealed duodenal perforations. One other animal had a sealed duodenal perforation, four had a dilated bile duct (without evidence of free perforation) and two had multiple small diverticula in the duodenum. No effects were seen in the stomach or major blood vessels (aorta, vena cava and portal vein).

Histologically, the treated tumours showed zones of necrosis, often haemorrhagic in the centre, sharply demarcated from adjacent viable tumour or normal pancreas and up to 12 mm in diameter with an inflammatory infiltrate in the surrounding area (Figure 3).

### DISCUSSION

This study has shown that it is possible to produce zones of necrosis up to 12 mm in diameter in tumours in the hamster pancreas using PDT with mTHPC. The main complication was sealed perforation of the duodenum, which was seen in 3 of 16 tumour-bearing animals treated. This is a much lower incidence than was seen in the earlier experiments treating normal tissues in the region of the pancreas (Mlkvy et al, 1996) and is mainly as a result of shielding the duodenum during treatment. The perforations that did occur may have been caused by slipping of the opaque paper shielding the duodenum, particularly as two of the three perforations occurred in animals treated with fractionated light, hence the treatment times were longer. The human duodenum is much thicker than that in the hamster and is likely to be much more resistant to perforation. Two preliminary clinical reports using PDT with Photofrin to treat duodenal and ampullary tumours showed an encouraging response with no perforations (Abulafi et al, 1995; Mlkvy et al, 1995). This suggests that it is likely to be safe to treat lesions in the human pancreas. Bile duct obstruction was seen in four animals but our previous studies showed that this was resolved by 7 days (Mlkvy et al, 1996); there were no perforations and hence it was most likely to be due to ampullary oedema. If this occurred clinically, it could be relieved by endoscopic insertion of a biliary endoprosthesis. The diverticulosis of the duodenum seen in two animals was unexpected, perhaps caused by partial obstruction of the distal duodenum, but had no obvious undesirable consequences. The sealed duodenal perforations in addition to three laparotomies within a month probably contributed to the death of two animals before the planned date of sacrifice. It might have been expected that PDT would cause an acute pancreatitis and perhaps even lead to pancreatic cyst formation, but we saw no evidence of this. The animals in this study were only kept alive for a few days but, even in our previous work (Regula et al, 1994) and in that of others (Evrard et al, 1994) in which animals were kept alive for up to 3 months, no evidence of pancreatitis or cyst formation was seen.

Other publications (Mang and Wiemann, 1987; Schroder et al, 1988; Chatlani et al, 1992; Evrard et al, 1994; Regula et al, 1994) have demonstrated pancreatic tumour necrosis with PDT using the photosensitizing agents AlSPc, Photofrin, ALA and pheophorbide A. As the tumour model used here has been shown to share many characteristics with the human disease (Takiyama et al, 1990), our results are encouraging for the application of PDT to human pancreatic cancers. Photofrin has the longest duration of skin photosensitivity, which can last for months, in contrast to just 1–2 days with ALA. AlSPc and pheophorbide A cause few skin problems in animals (Tralau et al, 1989; Evrard et al, 1994), but there are no clinical data; mTHPC does make patients photosensitive for 2–3 weeks.

Several factors determine the depth of necrosis produced; these include the dose of photosensitizing drug, the light dose and the light delivery geometry. Another is the wavelength of light used. AlSPc has a strong absorption peak at 675 nm, giving better light penetration of tissue than is possible at shorter wavelengths. Pheophorbide A and mTHPC absorb less strongly at 665 nm and 652 nm, respectively, whereas ALA/PPIX and Photofrin require excitation at 630–635 nm, which corresponds to relatively weak absorption peaks. It is difficult to compare the published results with all these photosensitizers as the geometry of the light delivery systems used and the ways of quantifying tissue necrosis differed, but ultimately the aim is to destroy as much tumour as possible with the minimum number of treatment sites and no unacceptable damage to adjacent normal tissues. The maximum diameter of the zone of necrosis in tumour with light delivered via a single fibre touching the tumour surface (as used for ALA, AlSPc and mTHPC) was greatest using mTHPC (up to 12.4 mm). This was achieved using light fractionation and, using continuous light, the value (8.5 mm) was similar to that found with ALA (8 mm, Regula et al, 1994) and AlSPc (8 mm, Chatlani et al, 1992). The

© Cancer Research Campaign 1997

choice of treatment time after administration also varies considerably between these sensitizers: 3–4 h for ALA/PPIX compared with up to several days for mTHPC. We compared the efficacy of mTHPC at 2 and 4 days and found that larger lesions were present for 4 days in agreement with the results of the fluorescence microscopy studies. However, the correlation between PDT efficacy and sensitizer pharmacokinetics, whether measured using fluorescence microscopy or radiolabelling, should not be overstated as the microscopic distribution of the sensitizer will vary after administration, with differing amounts present in tumour compartments (Chatlani et al, 1992); differential uptake by macrophages may also be an important factor. Even if similar mean concentrations are found at different times after administration, this does not necessarily translate into similar PDT efficacy as the microscopic distributions may be significantly different.

So far, only one randomized, controlled survival study has been reported (Regula et al, 1994), but this did show a significantly increased survival time for hamsters with transplanted pancreatic cancers treated with PDT using ALA compared with untreated controls. Although the study from Evrard et al (1994) using Pheophorbide A was not randomized, their animals also survived longer than would have been expected from historical controls. The present work did not include a survival study, but as the diameter of PDT necrosis in these hamster cancers using mTHPC with a single-fibre treatment point was at least as large as that in the ALA study, it is likely that a survival advantage could be shown.

Much has been written about the selectivity of uptake of photosensitizers in malignant tissue. In most tissues, the ratio of photosensitizer concentration in tumour to that in the adjacent normal tissue in which the tumour arose rarely rises above 2–3:1, and it is difficult to get any tumour necrosis without damage to adjacent normal tissue if both are exposed to the same light dose (Bown, 1990). The highest ratio of photosensitizer concentration between pancreatic cancer and normal pancreas reported, i.e. 13.5, is with pheophorbide A (Evrard et al, 1994). The figures for the other photosensitizers are 8:1 for protoporphyrin IX (the active derivative of ALA) obtained by Regula et al (1994), but only 1.2:1 in the present study with mTHPC using the same transplanted tumour model. A ratio of 3:1 for AlSPc (Chatlani et al, 1992) and Photofrin (Schroder et al, 1988) was obtained using a chemically induced autochthonous pancreatic tumour model. An interesting finding common to all studies is that normal pancreas appears to be relatively resistant to PDT. It has been postulated that there may be a singlet oxygen scavenger in normal pancreas (perhaps glutathione) that is not present in pancreatic cancers and that protects the normal areas from PDT damage (Chatlani et al, 1992). If this different mechanism for therapeutic selectivity is true, it could be possible to obtain selective tumour necrosis even without higher levels of the photosensitizing agents in tumour. In our studies using 0.3 mg $kg^{-1}$ mTHPC and a continuous light dose of 50 J delivered via a single fibre just touching the tissue surface, the diameter of necrosis in normal pancreas was 4 mm (Mlkvy et al, 1996) compared with 8.5 mm in tumour, as reported in this paper.

Fractionation of the light dose used for PDT is attracting increasing interest. Two reports described enhancement of PDT necrosis of animal tumours using ALA (van der Veen et al, 1994) and HpD (Pe et al, 1994) with treatment breaks of 60–90 min between fractions. More dramatic effects were described by Messman et al (1995) using ALA with intervals of only a few minutes between light fractions; they showed that under appropriate circumstances, the area of necrosis in normal colon could be increased by a factor of 7. Hua et al (1995) achieved a significantly increased tumour (transplanted rat mammary adenocarcinoma) doubling time using ALA when the light dose was modulated in a 30 s on/off protocol. Using mTHPC or Photofrin, van Geel et al (1996) found that only certain fractionation schedules were effective in limiting regrowth of a RIF-1 tumour model: for mTHPC, discontinuous irradiation with the 30 s on/off protocol proved effective at a fluence rate of 100 mW $cm^{-2}$. The most likely mechanism is that the dark intervals are permitting reoxygenation of the partly treated tissue. In our previous study with mTHPC on normal pancreas, we obtained an increase in lesion size of about 30% by dividing the light dose into four fractions separated by 1-min intervals (Mlkvy et al, 1996). Our current work on a tumour model has shown that, with a dose of 0.3 mg $kg^{-1}$ mTHPC 4 days before PDT with a total light dose of 50 J, the average diameter of necrosis could be increased from 8.5 mm with continuous light to 12.1 mm with fractionation, an increase of about 40%. This could perhaps be improved further by changing the point during treatment at which the break is made and the duration of the break.

Few studies have been undertaken of PDT in other solid rather than hollow organs, but recent reports of experiments in the normal canine prostate show that zones of necrosis up to 24 mm in diameter can be produced around single fibres placed interstitially after photosensitization with mTHPC (Chang et al, 1996). Using AlSPc, the maximum lesion size in the prostate was only 12 mm (Chang et al, 1997). From our clinical studies on tumours of the mouth (unpublished data), it is becoming clear that deep PDT effects can be achieved with remarkably low light doses (5–20 J $cm^{-2}$), which further indicates that mTHPC is a valuable photosensitizer for producing larger volumes of necrosis with low light doses and, consequently, short treatment times.

These studies suggest that PDT is a safe technique for treating cancers in the pancreas with any of the photosensitizers discussed here. Selectivity of tumour uptake was least using mTHPC, but this is not critical. The attraction of mTHPC is the possibility of producing larger volumes of necrosis around each treatment site with low light doses and hence shortening treatment times. In patients with small cancers localized to the pancreas who are unfit for pancreatectomy because of their general medical condition and for whom there are no other therapeutic options, it would now seem justified to consider pilot clinical studies if the free flow of bile is protected by a biliary endoprosthesis. Laser fibres could be positioned in the tumour through needles placed percutaneously under ultrasound or computerized tomography (CT) guidance, as is done routinely in the management of small liver tumours (Amin et al, 1993), and the results assessed by contrast-enhanced CT scans.

### ACKNOWLEDGEMENTS

Dr Peter Mlkvy was funded by The Association for International Cancer Research. m-THPC was donated by Scotia Pharmaceuticals, Guildford, UK.

### REFERENCES

Abulafi AM, Allardice JT, Williams NS, van Someren N, Swain CP and Ainley CA (1995) Photodynamic therapy for malignant tumours of the ampulla of Vater. *Gut* **36**: 853–856

Amin Z, Donald JJ, Masters A, Kant R, Steger AC, Bown SG and Lees WR (1993) Hepatic metastases: interstitial laser photocoagulation with real-time ultrasound monitoring and dynamic CT evaluation of treatment. *Radiology* **187**: 339–347

© Cancer Research Campaign 1997 

Berenbaum MC, Akande SL, Bonnett R and Kaur H (1986) MesoTetra (hydroxyphenyl) porphyrins a new class of potent tumour photosensitiser with favourable selectivity. *Br J Cancer* **54**: 717–725

Berenbaum MC, Bonnett R, Chevretton EB, Akande-Adebakin SL and Ruston M (1993) Selectivity of meso Tetra (hydroxyphenyl) porphyrins and chlorins and of Photofrin II in causing photodamage in tumours, skin, muscle and bladder. The concept of cost benefit in analysing results. *Lasers Med Sci* **8**: 235–243

Bonnett R, White RD, Winfield UJ and Berenbaum MC (1989) Hydroporphyrins of the meso-tetra(hydroxyphenyl) porphyrin series as tumour photosensitisers. *Biochem J* **261**: 277–280

Bown SG (1990) Photodynamic therapy to scientists and clinicians – one world or two? *J Photochem Photobiol B: Biol* **6**: 1–12

Chang S-C, Buonaccorsi G, MacRobert A and Bown SG (1997) Interstitial and transurethral photodynamic therapy of the canine prostate using meso-tetra-(m-hydroxyphenyl) chlorin. *Int J Cancer* **67**: 555–562

Chang S-C, MacRobert AJ and Bown SG (1996) Interstial photodynamic therapy of canine prostate with 5-aminolaevulinic acid and sulphonated aluminium phthalocyanine. *Prostate* (in press.)

Chatlani PT, Nuutinen PJO, Toda N, Barr H, MacRobert AJM, Bedwell J and Bown SG (1992) Selective necrosis in hamster pancreatic tumours using photodynamic therapy with phthalocyanine photosensitisation. *Br J Surg* **79**: 786–790

Chevretton EB, Berenbaum MC and Bonnett R (1992) The effect of PDT on normal skeletal muscle in an animal model. *Lasers Med Sci* **7**: 103–110

Dilkes MG, DeJode ML, Rowntree-Taylor A, McGilligan JA, Kenyon GS and McKelvie (1996) m-THPC photodynamic therapy for head and neck cancer. *Lasers Med Sci* **11**: 23–29

Dougherty TJ, Cooper MT and Mang TS (1990) Cutaneous phototoxic occurencies in patients receiving Photofrin. *Las Surg Med* **10**: 485–488

Egami H, Takiyama Y, Cano M, Houser WH and Pour PM (1989) Establishment of pancreatic ductal carcinoma cell line (PC-1) producing blood group-related antigens. *Carcinogenesis* **10**: 861–869

Evrard S, Keller P, Hajri A, Balboni G, Mendoza-Burgos L, Damge C, Marescaux J and Aprahamian M (1994) Experimental pancreatic cancer in the rat treated by photodynamic therapy. *Br J Surg* **81**: 1185–1189

Hua Z, Gibson SL, Foster TH and Hilf R (1995) Effectiveness of 5-aminolaevulinic acid-induced protoporphyrin as a photosensitiser for photodynamic therapy in vivo. *Cancer Res* **55**: 1723–1731

Mang TS and Wieman TJ (1987) Photodynamic therapy in the treatment of pancreatic carcinoma: dihematoporphyrin ether uptake and photobleaching kinetics. *Photochem Photobiol* **46**: 853–858

Messmann H, Mlkvy P, Buonaccorsi G, Davies C, MacRobert AJ and Bown SG (1995) Enhancement of photodynamic therapy with 5-aminoalaevulinic acid induced porphyrin photosensitisation in normal rat colon by threshold and light fractionation studies. *Br J Cancer* **72**: 589–594

Mlkvy P, Messmann H, Debinski H, Regula J, Conio M, MacRobert AJ, Spigelman A, Phillips R and Bown SG (1995) Photodynamic therapy for polyps in Familial Adenomatous Polyposis – a pilot study. *Eur J Cancer* **31A**: 7/8, 1160–1165

Mlkvy P, Messmann H, Pauer M, Stewart JCM, Millson CE, MacRobert AJ and Bown SG (1996) Distribution and photodynamic effects of meso-tetrahydroxyphenylchlorin (m-THPC) in the pancreas and adjacent tissues in the Syrian golden hamster. *Br J Cancer* **73**: 1473–1479

Nuutinen PJO, Chatlani PT, Bedwell J, MacRobert AJM and Bown SG (1991) Distribution and PDT effect of disulphonated aluminium phthalocyanine in the pancreas and adjacent tissues in the Syrian golden hamster. *Br J Cancer* **64**: 1108–1115

Pe BM, Ikeda H and Inokuchi T (1994) Tumour destruction and proliferation kinetics following periodic, low power light, haematoporphyrin oligomers mediated photodynamic therapy in the mouse tongue. *Oral Oncol Eur J Cancer* **30**: 174–178

Ravi B, Regula J, Buonaccorsi GA, MacRobert AJ, Loh CS and Bown SG (1996) Sensitization and photodynamic therapy of normal pancreas, duodenum and bile ducts in the hamster using 5-aminolaevulinic acid. *Lasers Med Sci* **11**: 11–21

Regula J, Ravi B, Bedwell J, MacRobert AJM and Bown SG (1994) Photodynamic therapy using 5-aminolaevulinic acid for experimental pancreatic cancer – prolonged animal survival. *Br J Cancer* **70**: 248–254

Ris HB, Altermatt HJ, Inderbitzi R, Hess R, Wachbur B, Stewart JCM, Wang Q, Lim CK, Bonnett R and Berenbaum MC (1991) Photodynamic therapy with chlorins for diffuse malignant mesothelioma: initial clinical results. *Br J Cancer* **64**: 1116–1120

Ris HB, Altermatt HJ, Nachbur B, Stewart CJM, Wang Q, Lim CK, Bonnett R and Althaus U (1993) Effect of drug-light interval on photodynamic therapy with meta-tetrahydroxyphenylchlorin in malignant mesothelioma. *Int J Cancer* **53**: 141–146

Savary JF, Monnier P, Fontolliet C, Wagniere JG, Braichotte D and Van den Bergh H (1995) mTHPC, a second generation photosensitizer for PDT of early squamous cell carcinomas of the oesophagus, bronchi and mouth (abstract VI-4s/04). European Society for Photobiology 6th Congress, September 1995, Cambridge, UK

Schroder T, Chen IW, Sperling M, Bell RH Jr, Brackett K and Joffe SN (1988). Hematoporphyrin derivative uptake and photodynamic therapy in pancreatic carcinoma. *J Surg Oncol* **38**: 4–9

Takiyama Y, Egami H and Pour PM (1990) Expression of human tumour-associated antigens in pancreatic cancer in Syrian golden hamsters. *Am J Pathol* **136**: 707–715

Tralau CJ, Young AR, Walker NPJ, Vernon DI, MacRobert AJ, Brown SB and Bown SG (1989) Mouse skin photosensitivity with dihaematoporphyrin ether (DHE) and aluminium sulphonated phthalocyanine (AlSPc): a comparative study. *Photochem Photobol* **49**: 305–312

Van der Veen N, van Leengoed HLLM and Star WM (1994) In vivo fluorescence kinetics and photodynamic therapy using 5-aminolaevulinic acid-induced porphyrin: increased damage after multiple irradiations. *Br J Cancer* **70**: 867–872

Van Geel IPJ, Oppelaar H, Marijnissen JPA and Stewart FA (1996) Influence of fractionation and fluence rate in photodynamic therapy with Photofrin or mTHPC. *Radiat Res* **145**: 602–609

 © Cancer Research Campaign 1997